# Ethical considerations for children’s participation in data collection activities during humanitarian emergencies: A Delphi review

**DOI:** 10.1186/s13031-017-0108-y

**Published:** 2017-03-27

**Authors:** Cyril Bennouna, Hani Mansourian, Lindsay Stark

**Affiliations:** 1 Center on Child Protection and Wellbeing (PUSKAPA), School of Social and Political Sciences (FISIP), University of Indonesia, Gedung Nusantara II, Lantai 1, Depok, West Java 16424 Indonesia; 2 Program on Forced Migration and Health (PFMH), Mailman School of Public Health, Columbia University, 60 Haven Avenue, B-4 Suite 432, New York, NY 10032 USA

**Keywords:** Research ethics, Child participation, Child protection, Humanitarian, Emergency, Delphi

## Abstract

**Background:**

Children’s right to participate in data collection during emergencies has been widely recognized by humanitarian actors. However, participation in such activities can expose children to risk. Tensions have been noted between the right to participate and other principles, such as the imperative to ‘do no harm.’ With little evidence to inform guidance on addressing this tension, our study sought to identify expert consensus on whether and how children participate in emergency-related data collection activities.

**Methods:**

We employed a three-round Delphi technique with a purposive sample of 52 child protection specialists. Respondents answered two open-ended questions in round one. A thematic analysis of responses generated a set of unique statements addressing the study questions. In the second round, respondents rated each statement on a five-point scale. In the final round, respondents reviewed the group’s average ratings for each statement with the option to revise their own ratings. A statement was said to have reached clear consensus when at least 90% of respondents agreed or strongly agreed with the statement.

**Results:**

A total of 124 statements and 14 themes emerged from the thematic analysis, with 46.0% of statements reaching clear consensus in the third round. Respondents strongly supported children’s right to participate in data collection in humanitarian settings, while also recognizing that protecting children from harm may “over-ride” the participation principle in some contexts. Respondents identified capacity and contextual considerations as important factors influencing participation decisions, though they sometimes disagreed about how these factors should determine participation. Respondents also considered the role of individual child factors and the presence of caregivers in selecting child participants, and proposed best practice approaches for securing children’s safe and meaningful participation.

**Conclusions:**

With almost half of statements reaching clear consensus, these findings reflect broad agreement within the sector about engaging children in data collection in emergencies. At the same time, points of ongoing debate around how to factor different risks into child participation decisions may indicate discordant practice. Further reflection is needed around how factors such as the phase of emergency, the existence of basic services, and cultural beliefs should influence whether and how children participate.

**Electronic supplementary material:**

The online version of this article (doi:10.1186/s13031-017-0108-y) contains supplementary material, which is available to authorized users.

## Background

The principle of child participation is a cornerstone of the United Nations (UN) Convention on the Rights of the Child (CRC). The principle holds that children (defined as those under the age of 18) have the right to express their views if they so choose, and that, in accordance with the children’s age and maturity, these views should be taken into account for all matters that affect them [1]. There is a substantial literature on the ethics of child participation in research, with growing attention to the ethics of researching children exposed to humanitarian crises and displacement [2–9]. Several UN agencies and relief organizations have issued guidelines or toolkits to support the engagement of girls and boys in all aspects of the emergency programming cycle, including data collection activities for evidence generation, such as emergency assessments, monitoring and evaluation, and research [1, 10–20]. Children may participate in data collection as respondents or collaborators on the design and execution of data collection activities; they may also consult on the analysis, validation, or dissemination of the results [11]. This study focuses primarily on the participation of children as respondents in emergency-related data collection activities.

While child participation guidelines tend to recognize the value of including children in data collection activities, they also acknowledge that doing so can expose children to a host of physical and psychosocial risks, especially in conflict and disaster contexts, potentially creating tension between the principle of participation and other human rights and bioethics principles, namely the best interest of the child, respect for persons, non-maleficence (‘do no harm’), beneficence, and justice [7, 10, 21]. Emergencies can exacerbate the familiar constraints of resources, time, language capabilities, and insecurity during data collection, and the breakdown of critical infrastructure and social order can introduce innumerable additional obstacles. Simple tasks such as identifying secure data collection locations, establishing participants’ ages, and receiving consent from caregivers can become critical bottlenecks in the context of high mobility and ongoing conflict. By separating families and communities, and overwhelming child protection and other basic services, emergencies can also disrupt data collectors’ ability to act upon their participants’ acute needs. Worse still, many risks persist beyond data collection. Completed surveys, for example, can be seized by armed forces or groups and interpreted as condemning evidence, endangering both participants and enumerators. Even after investigators return safely home, respondents remain at risk of stigma and reprisal, sometimes complicating efforts to share research findings with participating communities [21].

Those conducting evidence generation activities in humanitarian settings must also negotiate what has been called the “dual imperative” between producing information capable of benefiting policies and programs during the response and generating high quality, scientifically valid results [22]. The former priority tends to be the most salient in the earliest stages of an emergency, when assessments aiming to determine the immediate needs of emergency-affected populations are the most common form of evidence generation. Due to the urgency of these assessments, they usually do not have the benefit of being reviewed by an Institutional Review Board (IRB) for ethical and legal suitability. According to the Inter-Agency Standing Committee, which coordinates humanitarian assistance across organizations globally, including emergency assessments, primary data collection should usually begin with a Multi Cluster/Sector Initial Rapid Assessment (MIRA) about 72 h after the onset of an emergency, though timing may vary according to the type of emergency and latent response capacity, and other data collection activities may sometimes precede the MIRA or take place concurrently [23]. The MIRA guidelines advise enumerators to take stock of children’s conditions in the direct observation component, and to speak face-to-face with affected people, including children among others, but do not include tools or principles for engaging children [23]. Sector-specific assessments, like the Child Protection Rapid Assessment (CPRA), should ideally begin in the third or fourth weeks following emergency onset and take several weeks to complete, though a global review found that most assessments did not begin until at least the eighth week following emergency onset [24, 25]. The CPRA guidelines recognize that children’s participation can contribute to a richer understanding of emergency situations, but do not recommend interviews or focus group discussions with children because “in most cases, it is unlikely that trained staff is available to conduct such highly sensitive interviews” [24]. Monitoring and evaluation efforts are tied to specific interventions, and may last the duration of emergency response.

Acknowledging the risks of engaging children in data collection during emergencies, the Committee on the Rights of the Child has reaffirmed that children’s right to participation “does not cease in situations of crisis or in the aftermath,” and it promotes children’s involvement in assessments and monitoring, among other activities [1]. Although the available literature on child participation provides a valuable inventory of techniques for engaging children meaningfully in data collection activities, and addresses core attending ethical concerns, such as the principles of beneficence, justice, and respect, considerable discordance remains among humanitarian practitioners and researchers about the specific factors that should determine whether and how children participate in data collection during the early stages of emergencies.

On behalf of the Child Protection Assessment, Measurement, and Evidence Working Group, which develops guidance, tools, and methodologies to improve evaluation capacities for child protection interventions in humanitarian settings, we utilized the Delphi technique to explore the diversity of perspectives among child protection specialists across the globe relating to this unresolved issue [26, 27]. The Delphi technique promotes reflection within a diverse panel of experts in a series of structured rounds to explore the possibility for agreement on certain thematic areas and to identify areas of ongoing debate. As opposed to informal consensus-building exercises, such as committee meetings, panel members remain anonymous in a Delphi study and do not interact with one another physically. This allows participants to reflect on their own time and in their own space, making it a practical method for involving humanitarian practitioners [28]. This characteristic is also thought to temper the biasing effects of dominant participants, social desirability, and other group dynamics [29]. For these reasons, researchers have used the Delphi technique to understand agreement and disagreement in a wide array of fields, including medicine, public health, disaster preparedness, and child protection [28–33].

The present study sought to elicit specialist opinions about the important factors that should determine whether and how children participate in data collection activities related to child protection during the early phases of emergencies. By exploring the degrees to which specialists agree on these factors, and locating points of ongoing disagreement, we expect that the study’s results will inform further deliberation regarding the development of child participation standards in humanitarian response.

## Methods

### Participants

We sampled members of the study panel purposively to represent at least one of the following three categories of expertise: (1) significantly experienced in measurement issues related to children in emergencies, (2) significantly experienced in child protection in emergency (CPiE) program design or policy development, and (3) currently providing guidance to field staff on assessment and measurement issues in emergency contexts. We developed a contact list of 82 individuals potentially meeting these criteria by holding consultations with members of the Alliance for Child Protection in Humanitarian Action (formerly known as the Child Protection Working Group or CPWG), scanning relevant organization websites, grey literature, and published literature, and by attending child protection sector conferences. In addition to meeting at least one of the above categories, individuals represented a wide distribution of experiences, institutional affiliations, and geographic origins, though all had to have a minimum proficiency in English. All individuals were initially contacted via email with an invitation to participate, a description of the study design and objectives, the Round I questionnaire, and a consent form.

### Procedure

The study employed a classic Delphi design with three successive rounds, beginning with free response elicitation in the first round and followed by two rounds of feedback and consensus-building [34–36]. Although additional rounds would be expected to result in greater consensus, we selected a three-round design to mitigate participant attrition, taking into consideration the challenges of repeated participation for field-based respondents, which constituted a sizeable proportion of the study sample [28]. We trialed the research instrument with a sample of 15 specialists prior to the first round, and the instrument was refined based on this pilot.

#### Round I

Those consenting to participate in the study were invited to answer a number of enrollment questions, followed by the main research questions, which asked respondents what conditions they believed should determine whether and how children are interviewed “as part of emergency assessments and other data collection activities in the early stages of an emergency,” and which guidelines they used, if any, to support their decisions about child participation. “Early stages” was defined as the onset and weeks following a conflict event or natural disaster. Involvement in “emergency assessments and other data collection activities” was defined as “the engagement of children in the direct provision of information to data collection teams in the early stages of emergencies for programming purposes.” The questionnaire instructed participants to respond in clear statements of whatever length they preferred and to return the completed form via email.

Following Round I data collection, our team collated the completed questionnaires and distilled them into a comprehensive list of unique statements [34]. The first author reviewed the full sample of completed surveys, identifying unique statements and compiling them into a master list. When one sentence consisted of multiple concepts or opinions, it was segmented into multiple statements, preserving the participant’s original wording to the extent possible. In the event that two participants expressed the same concept or opinion, one statement was generated to represent that idea using whichever participant’s wording was the clearest. The second and third authors independently reviewed the composite list against the completed surveys to ensure the statements were exhaustive of the unique ideas and opinions conveyed through the completed survey, to ensure clarity of wording, and to remove conceptual redundancies between statements.

Once we agreed on the finalized version of the statements list, we reviewed the list independently using inductive thematic analysis [28, 37]. This involved analyzing each statement with reference to the others, identifying thematic linkages between conceptually similar statements, and then grouping statements into the emergent themes. We then compared our initial sets of themes with one another and came to consensus on a final set. Lastly, we independently sorted each statement into a theme before reconvening and coming to consensus on a final master list of statements organized by theme.

#### Round II

We converted an anonymized version of the master statements list into a survey for Round II and emailed it to all participants in addition to nine participants that were unable to participate in Round I but consented to participate in subsequent rounds. Participants were instructed to rate each statement on a 5-point Likert scale from ‘strongly agree’ (rating of 5) to ‘strongly disagree’ (rating of 1). We also provided participants with an open-ended section to elaborate on their opinions.

#### Round III

In this final round, we sent participants the master statement list with their Round II ratings alongside the average ratings from the full study sample for each statement. They were then instructed to compare their own ratings against the group mean for each statement and to either confirm or modify their Round II rating.

### Final analysis

We defined “clear consensus” as any statement with which at least 90% of participants either agreed or strongly agreed [28]. Statements for which between 80% and 89% of participants agreed or strongly agreed were said to be approaching consensus. To provide a richer understanding of the polarity of opinions, we also calculated intensity ratios for each statement. Following Ager, Stark, Akesson, and Boothby, we defined “agreement intensity” (AI) as the proportion of participants agreeing with a statement that strongly agreed with it [28]. A score above 0.5 indicated that participants were more likely to agree strongly with the statement than agree moderately with it. “Disagreement intensity” (DI) was defined as the proportion of participants not agreeing with a statement (strongly disagree, disagree, or undecided) that either strongly disagreed or moderately disagreed with it. We included participants who were undecided in the disagreement intensity calculation because their scores detracted from consensus, even if they did not actively disagree with the statement. A score above 0.5 indicated that participants were more likely to disagree actively with the statement than simply feel undecided about it.

### Ethical considerations

Columbia University’s Institutional Review Board reviewed the study’s protocol and determined it to be exempt under IRB-AAAQ0600.

## Results

In total, 52 (37 female, 15 male) respondents participated in the study (see Table 1). Forty-three participated in Round I, resulting in 124 unique statements and 14 themes, followed by 46 respondents in Round II, and 42 respondents in Round III [see Additional file 1]. Respondents included specialists in child protection programming, policy development, and monitoring and evaluation, as well as social scientists, epidemiologists, psychologists, and donor representatives focusing on child protection in emergencies.

**Table 1:** Delphi participant characteristics

	Female	Male	Total
All Participants	37	15	52
Region of Origin			
*Africa*	2	5	7
*Australasia*	1	0	1
*Europe*	19	2	21
*Middle East/Central Asia*	1	0	1
*North America*	10	6	16
*South/East Asia*	5	2	7
Affiliation at Time of Participation			
*Humanitarian agency/organization*	25	12	37
*Academic researcher*	6	2	8
*Policymaker/Donor*	2	1	3
*Consultant*	4	0	4

The distribution of ratings was positively skewed, with a median score of four (indicating moderate agreement) in both rating rounds, and averages of 3.8 (standard deviation = 1.1) and 3.9 (standard deviation = 1.0) in rounds two and three, respectively. In the second round, 29.8% of statements achieved clear consensus, with an additional 24.2% approaching consensus. In the third round, clear consensus increased by more than half to 46.0% of statements (see Table 2), with another 13.7% approaching consensus (see Table 3).

**Table 2:** Delphi statements reaching clear consensus

Number	Statement	Consensus	AI	DI
S11	Children have a right to decide whether to participate in evidence generation. A child’s decision not to participate must be respected.	100%	0.98	0.00
S99	Data collection activities should be conducted in a language that child participants understand and are comfortable with.	100%	0.83	0.00
S59	Expectations need to be managed appropriately and transparently. Children should be informed about the data collection process before, during, and after the assessment. They should be told what will happen to the information and when and what they can expect.	100%	0.80	0.00
S85	While all children may lack power in relation to adults there are also power imbalances amongst children. These can be due to cross-cutting factors of age, gender, ethnicity, class-caste, etc. Care is needed to avoid silencing of some children by their more powerful peers.	100%	0.78	0.00
S94	We need to be thinking a lot more about adapting questions for different age groups – not just how we ask the questions but what we want to know.	100%	0.73	0.00
S93	Data collection activities should consider different methods to collect data from children, and these should be simple, age-specific, and culturally-adapted (e.g. focus group discussions, questionnaires, individual interviews, safety maps, drawings, etc.).	100%	0.68	0.00
S96	Data collection activities need to recognize and accommodate the other demands on children’s time in the context (e.g. are we taking time from children who would otherwise be generating income, caring for younger siblings, or attending food distributions?).	100%	0.59	0.00
S108	Those conducting data collection must be well-trained to collect data from children, must be familiar with available standards, and should be experienced in working with children directly.	100%	0.59	0.00
S102	The setting of data collection activities must be considered before involving a child in data collection, taking into account the child’s preference and the potential risks to the child and researcher of conducting the interview. For example, children may prefer to be interviewed in their home, or it may be better to interview them in child-friendly spaces or schools.	100%	0.56	0.00
S121	If indirect methods need to be used to assess children’s needs, or if exclusion of children from assessments is inevitable, the resulting report should note the absence of these children from the data and the implications of not having this information.	100%	0.56	0.00
S120	Feeding back information and results to children needs to be done in a child-friendly, simple manner, including if possible the visualization of information.	100%	0.54	0.00
S113	When recording the information provided by children it is crucial that the data collector is familiar with cultural norms and local customs to correctly record and interpret information provided by children using the local language, paraphrases, and typical local references.	100%	0.34	0.00
S15	Before any data collection is conducted with children, a risk analysis should take place, taking into account the political context, security situation, degree of stability/volatility, cultural factors, power dynamics, exclusion issues, the impact of the humanitarian context on these, and the risks posed by the data collection process itself. This risk analysis will inform decision making about when and how children’s participation may or may not be appropriate.	98%	0.73	0.00
S55	The child must have the psychological, cognitive and emotional ability to participate in data collection.	98%	0.27	1.00
S60	Children should be made aware that no one will be punished or receive any less help than anyone else if they do not participate – and likewise they should be told that neither they nor anyone else is going to get a reward or receive extra of anything for agreeing to participate.	98%	0.88	1.00
S110	Those conducting data collection need to be trained in recognizing any signs of distress demonstrated by children, need to be able to provide any immediate response required (reassure, record) and be able to assess the need to terminate the data collection appropriately.	98%	0.70	0.00
S62	If using a digital device to record a child’s participation, researchers should assess if this is appropriate (e.g. for security reasons or cultural belief), and if both children and parents are comfortable using the device.	98%	0.53	1.00
S114	Analysis teams for data that came from children need to value and be able to interpret a variety of types of evidence (e.g. visual as well as text-based).	98%	0.48	1.00
S86	Clear and transparent criteria for inclusion/exclusion of children in evidence gathering processes should be developed and should reflect considerations of participant safety, cultural acceptability, and appropriateness for evidence gathering.	98%	0.45	0.00
S95	Data collection instruments for children should undergo careful cognitive and field testing prior to implementation to ensure that minors will understand the questions/activity, to identify implementation challenges, and to assess risks and impacts on children of using the instrument.	98%	0.40	0.00
S92	In all contexts assessment team members should observe the situation of girls and boys (of different ages and abilities), including observation and recording the roles and responsibilities undertaken by girls and boys of different ages and backgrounds.	98%	0.38	0.00
S28	There should be a mechanism with clear protocols for following up on urgent issues and/or resolving pressing issues that have come to light.	98%	0.87	1.00
S98	There should be sufficient time for children of different ages to formulate their ideas, react to information, react to each other, and adequately discuss and be heard.	98%	0.41	0.00
S101	Child-friendly interpretation must be provided for any interviews or activities with children (e.g. it is not enough that someone has linguistic skills; they must appreciate the additional issues involved in interpreting for children).	98%	0.36	0.00
S103	If the child is being interviewed alone, clear SOPs are needed, and the regulations, local cultural beliefs, and laws need to be respected.	97%	0.42	0.00
S14	The principles of respect, beneficence (harm and benefits), and justice should underpin all assessment activities, with children and adults.	95%	0.85	0.50
S12	The quality of the participatory process is important for children’s experience of data collection activities.	95%	0.83	0.00
S6	Child rights and humanitarian principles, including the principle of the child’s best interests and the principle of ‘do no harm’ may over-ride the principle of children’s participation in some contexts.	95%	0.75	1.00
S20	Cultures where the expectation is that children do not talk, or do not talk when adults are present, or do not talk about certain issues need to be understood and carefully negotiated by contact with adult ‘gatekeepers’ so that children do not face retaliation, forms of punishment, or other negative consequences as a result of their participation. Children need to be approached through the ‘proper channels’ in context.	95%	0.53	0.00
S9	In most societies, children lack power in relation to adults. This makes data gathering directly with children especially important since their views can be marginalized and/or misrepresented by adults.	95%	0.50	0.00
S58	Informed consent is not a one-off event, it is an ongoing process, which should be re-evaluated, depending on changes in the circumstances of the emergency and other contextual factors.	95%	0.79	0.00
S89	Since the effects of emergencies and the needed supports are gendered, one cannot defend the common practice of interviewing boys more than girls. It is crucial to learn from girls and engage with them as actors and participants who are agents of their own protection and well-being.	95%	0.51	0.50
S56	Children should only participate in data collection once they have provided informed consent/assent, and when needed, their care givers have also consented.	95%	0.49	1.00
S109	It is essential in training to ensure that staff demonstrate their skills in engaging children’s participation in data collection, and that only those who perform well be selected to conduct the activities.	95%	0.44	1.00
S111	The gender of the researchers capable of collecting data also matters in deciding if and which children to include in the data collection design.	95%	0.33	0.00
S81	Children should be offered privacy, without their participation drawing undue or unwanted attention and thus potentially singling them out.	95%	0.28	0.00
S49	After the initial 4-6 weeks of the sudden onset emergency response, opportunities for children’s participation in programme planning and implementation start to increase in contexts where the situation has become more stable. Especially in contexts where children and families are living in their own communities, and/or in established refugee or IDP camps, there may be increased opportunities for community based work and regular interactions with children and community members providing a basis for meaningful participatory processes supporting collaborative and/or child led initiatives.	95%	0.28	0.00
S21	The presence of a protective environment/support network needs to be in place when discussing more sensitive child protection issues such as sexual violence, child soldiers, etc.	95%	0.73	0.00
S38	I think children should be interviewed as part of family tracing work or care and protection proceedings to ensure that decisions are made in their best interests considering their own views.	93%	0.72	0.00
S10	Children can be respondents but also advisors, researchers, advocates, analysts, documenters. But this will depend on providing them with the necessary skills and information to be partners in this as well as applying the basic requirements for their ethical and meaningful involvement.	93%	0.59	0.33
S84	Confidentiality and protection of personal data should be guaranteed to all children.	93%	0.95	0.67
S106	Adult investigators enjoy a position of power in relation to children. The humanitarian context can exacerbate the power differential between investigators and children. Ethical data collection entails working to minimize power differentials by, for example, sitting at the same level as children, participating in games, showing careful attention to the views expressed and showing appreciation for each individual’s contribution.	93%	0.66	0.00
S80	The presence of the legal guardian or another trusted person during data collection might be necessary to put the child at ease, but it should be carefully weighed against any possible bias.	93%	0.24	0.00
S118	Children should receive feedback on the findings of an assessment, and should be able to provide ongoing reflections and ask further questions after data collection has stopped.	93%	0.32	0.00
S13	Children should not participate in data collection if doing so presents added child protection risks.	90%	0.74	1.00
S2	In principle, it is important to consult with children on matters affecting them – thus children should be a source for assessments that will influence humanitarian planning and budgeting.	90%	0.47	0.50
S1	Providing children the opportunity to express their experiences is a powerful exercise, allowing them to release their feelings and make their voices heard.	90%	0.47	0.75
S23	Depending on the subject, it may be more appropriate to ask children about the experiences of children in their community, rather than about their own experiences.	90%	0.29	0.25
S22	Consulting with child protection experts experienced with the local context, and other community members, such as teachers, is important for deciding what subjects can be discussed with children by age-group and what topics should be avoided.	90%	0.29	0.25
S43	It is often more appropriate to secure data on younger children during the early stages of emergencies by other means than direct interviews – whether through a caregiver or resilient existing support networks within the community. For older children, it may be appropriate to engage directly from the onset of assessment and design.	90%	0.11	0.25
S123	We should not be establishing a norm ‘for’ or ‘against’ participation, but should be providing guidance on when participation is useful, under what circumstances, who it should be done by and how.	90%	0.65	0.50
S119	Feeding back to adults the findings from discussions with children could in itself be a powerful intervention. At the same time, the asymmetry of power means that issues raised by children in data gathering that call into question the actions of adults need to be addressed with sensitivity in order to avoid any potential backlash against participants.	90%	0.32	0.00
S34	Multi-sector initial rapid assessments are critical to defining the needs and vulnerabilities of children and establishing a program strategy that addresses these holistically through integrated programming. Thus, wherever risk assessments identify a safe way to consult and involve girls and boys, there are likely to be significant benefits of interviewing them to better understand their experiences and priorities.	90%	0.30	0.50
S33	If a humanitarian agency has existing programming or partnerships that support children’s participation then the scope for children’s participation is much greater from the outset of an emergency response, and there may also be safe and meaningful opportunities to collaborate with children and young people in undertaking an emergency assessment.	90%	0.24	0.00
S88	Clan or tribal associations matters during assessments, especially if a child is isolated from others.	90%	0.14	0.25
S107	There should be a core set of criteria that lists the skills and knowledge that a researcher should possess in order to involve children directly in data collection activities, and these should be standardized across organizations and institutions.	90%	0.36	0.00
S35	In situations of extreme violence or devastation, it is important to consider whether, given what children have been through, it may be better to not conduct individual interviews with children immediately, but rather to first establish psychosocial and other services and learn more about children’s needs through service provision.	90%	0.31	0.50

**Table 3:** Delphi statements approaching consensus

Number	Statement	Consensus	AI	DI
S115	It would be unhelpful and unethical to ask children questions without having a clear data management strategy. Children may easily begin to tell the story of what they have experienced and continue to experience, but this is qualitative data which is notoriously costly and time-intensive to manage. A clear structure to consultations with children is required.	88%	0.53	0.60
S100	In general but especially for younger children, data collectors should use the interviewee’s language/terms. If it is not clear to the interviewer what is being said, they should ask the child ‘What do you call _?’ using names rather than pronouns, use simple and short sentences, avoiding questions involving time, rephrasing questions, not repeating them, establishing a space where only stories are told that happened, no lying, etc.	88%	0.39	0.20
S124	The challenge is when an inter-agency approach is required and different approaches to programming may create a clash on assessment methodology. This is why one tool cannot meet the requirements of all agencies at all stages of the emergency. It is more helpful to have a toolkit where inter-agency child protection actors can be guided to develop a customized approach, based on standardized tools matched with the current reality.	88%	0.36	0.20
S112	In data collection activities with children in conflict settings of a political or ethnic nature, enumerators should either be selected based on their neutrality or based on their membership to the same ethnic, political, or military group as the children.	88%	0.25	0.00
S51	It may be that it is less risky to engage with children in a natural disaster setting rather than conflict-based emergency. However, it is rarely that clear.	88%	0.11	0.60
S7	If children’s participation is primarily motivated by an effort to secure their rights to express their opinions (rather than trying to produce new information), it would be better for children to be given continuous opportunities to participate in the design and implementation of the concerned agency’s program.	86%	0.58	0.17
S26	A decision tree could help make an informed decision as to whether or not to include children in any given data collection effort. It could also help put in place additional safeguards and mitigation measures when necessary.	86%	0.14	0.33
S57	Children should only participate in data collection when they understand consent.	85%	0.60	0.50
S97	An interview with children should not take too long, an hour maximum.	85%	0.37	0.67
S74	Local beliefs and attitudes towards children should be weighed when deciding whether to involve children in data collection activities.	85%	0.14	0.33
S32	One guiding factor in deciding whether to involve children directly in data collection activities should be whether there is the intention to programme/deliver services for a certain issue. If not, then one is likely gathering data for the sake of it, which is broadly considered unethical, especially in early onset emergencies, or where resources are limited.	83%	0.59	0.43
S67	Children as of 10 years old would possibly be able to provide useful information regarding the humanitarian needs and response.	83%	0.18	0.14
S91	Consultations should be carried out separately with girls and boys of different age groups.	83%	0.12	0.29
S66	Children ages 5-12 may engage in meaningful research and programming if specialized research tools are used by a skilled research team.	80%	0.27	0.50
S36	Children’s participation in community based committees and in accountability mechanisms can be harnessed to identify and reach the most marginalized children and their families and to ensure that the humanitarian response is benefitting those who are most in need.	80%	0.24	0.38
S77	Older teenagers may not consider themselves children (and perhaps others in the community don’t either) so calling them children can be unhelpful at least and insulting and close doors at worst.	80%	0.18	0.50
S117	It is important to come back after data collection activities with children and review earlier findings through subsequent interviews so that researchers can check their assumptions, make sure their actions were appropriate, assess how the situation has changed since the initial data collection, and determine what these changes mean for programming.	80%	0.19	0.13

The ratings reflect broad consensus among child protection practitioners and researchers from various organizations and geographies on a range of themes related to the ethics of child participation in emergency-related data collection. In line with the CRC, participants agreed that, in principle, children have a fundamental right to participate in data collection during and immediately following emergencies, and that this right draws from children’s unique experiences and perspectives. Almost all respondents felt that children lack power in relation to adults, and that this dynamic, which risks excluding children’s interests from adult testimony, creates an imperative to include children in emergency data collection activities (95%, S9). Respondents largely agreed that, in addition to benefiting decision-making related to emergency programs and policies, participation in data collection activities could also directly benefit children by allowing them to express their views, needs, and experiences (90%, S1).

A large majority of respondents also felt, however, that participation in emergency data collection activities could present significant risks to children and that other principles, such as non-maleficence, could “over-ride” the principle of participation (95%, S6). Respondents reached clear consensus around the notion that the principles of respect for persons, justice, and beneficence should underpin all data collection activities with children and adults (95%, S14). There was less agreement about how to weigh the various risks while making decisions about children’s participation. The three broad areas of debate commonly discussed by respondents included: (1) *whether* to invite children to participate in data collection, (2) *how* to select child participants inclusively, and (3) *which* methods and techniques to employ with different children to ensure their safe and meaningful participation. Respondents identified several considerations influencing each of these decisions, and the following sections review the range of specialist opinions about how these should factor into decision-making.

### Should children participate in emergency assessments and related data collection activities?

Approximately two-thirds of respondents agreed that children should be given the opportunity to participate in data collection activities “in all circumstances,” as long as investigators adjust the method and degree of participation to the demands of the context (69%, S5). A sizeable minority, however, strongly disagreed (24%, DI = 0.77). As one dissenting respondent explained, “This seems like a big stretch. You’ll end up adjusting the level of participation for some situations so much that you dilute the concept of participation and it becomes meaningless.” According to this view, the insistence on children’s involvement can in some circumstances lead to tokenistic participation, degrading the value of participation and introducing its own set of risks.

Two thematic areas emerged that dictated whether participants felt that children should be excluded from direct participation in a given data collection activity. The first was the capacity of the institution and supporting organizations associated with the activity, which included concerns with staff qualification, the existence of child-focused services in the selected area, and the intentions and ability to act upon the activity’s findings. The second thematic area related to contextual considerations, including the type and phase of emergency, and an assessment of the underlying demographic and sociopolitical context of the selected area.

#### Institutional capacities

Respondents agreed unanimously that those collecting data from children should have specialized training in child-friendly methods and should be experienced using these methods (S108). Almost all respondents also agreed that investigators should be trained to recognize and respond to signs of distress during data collection (98%, S110) and that they should be asked to demonstrate their skills before collecting data from children (95%, S109). In addition to these qualifications, all respondents felt that investigators should be familiar with the participating children’s cultural norms and language (S113), and most agreed that, in accordance with the context and topic being investigated, the investigator’s gender (95%, S111), ethnicity, and political and military affiliations were important considerations (88%, S112). Taken together, these statements reflect broad agreement that data collection teams must have requisite cultural and professional competency before children are invited to participate. Indeed, 90% of respondents felt that data collectors should be required to meet standardized criteria of minimum skills and knowledge before engaging child participants (S107). Those who did not agree tended to question the feasibility of establishing a shared standard for demonstrated competencies. As one respondent remarked, “Standardization across organizations is not going to happen, ever. But [I] agree that a core set of skills and competencies would be helpful.”

Respondents reached clear consensus on the idea that the scope for child participation would be greater in cases where investigators were associated with supporting humanitarian programs and partnerships (90%, S33). Respondents also agreed that in all cases investigators should have a clear protocol for following up on urgent issues arising during data collection activities (98%, S28). Most respondents thought that children should be excluded from participation if data collectors lacked the time to address signs of distress (71%, S29), while many of those who did not agree explained that, rather than responding directly to distress, it was more important that data collectors be able to refer children to service providers. A majority agreed that the intention to deliver programs or services to children in response to data collected should guide whether the activity includes child participants, with the addendum that “gathering data for the sake of it” is “broadly considered unethical” (83%, S32). Those who disagreed explained that children’s participation could still be justifiable if the results helped to document needs and abuses, and especially if they could inform future programming priorities.

#### Contextual considerations

Almost all specialists agreed, often strongly, that in order to decide whether child participation is appropriate in a given study, investigators should conduct a risk analysis of social, political, security, and cultural factors (98%, AI = 0.73, S15). There was considerable ambivalence, however, about how the results of such an analysis could determine whether children participate. For example, 15% of respondents did not agree with a statement asserting that local beliefs and attitudes should be weighed when deciding whether to involve children (DI = 0.33, S74). As one respondent remarked, “but what if local beliefs tell us not to listen to children?” In fact, 10% of respondents felt that, “it may not be cost-effective to interview children in cultures that do not encourage children to speak up,” and another 10% were undecided (S76). Some respondents took issue with the particular approach of interviewing children, but many agreed that children simply should not participate in such situations. As one respondent explained, involving children in such cultures “might create additional tension following the assessment.” In a related statement, 95% of respondents agreed that in cultures where children are not expected to express themselves actively, data collectors should work with adult “gatekeepers” to ensure that children are not punished as a result of having participated in the activity (S20).

Aside from cultural considerations, almost a third of respondents felt that “the time required to consult with children and analyze these data may not be worth the investment in a rapidly changing context and where resources are limited” (27%, S8). Similarly, 30% of respondents thought that children should only be interviewed in emergency assessments when the data are not available from other sources (S4). Meanwhile, 48% of respondents disagreed with this sentiment, and an additional 10% disagreed strongly (DI = 0.82), arguing that, an “interview with adults and previous research can’t replace the perspectives of children in a given context.”

Respondents were also ambivalent about how the phase and type of emergency should factor into child participation decisions. Only two of 16 statements related to this theme reached clear consensus, with an additional statement approaching consensus (see S43 and S49 in Table 2 and S51 in Table 3). Just under two-thirds of respondents thought that the type and phase of emergency should be considered “major factors” in deciding whether to include children (64%, DI = 0.89, S39). There was near-consensus, on the other hand, on the notion that it would be less dangerous to engage children in data collection during natural disasters than in contexts of armed conflict, but commenters cautioned that the distinctions between these types of emergency were “rarely that clear” (88%, S51).

Importantly, respondents were divided when it came to the question of whether it would be appropriate to interview children in the early phases of emergencies, especially “where there is a significant level of uncertainty for the child, lack of basic services, or violence targeting children” (43%, S40). As one respondent commented, investigators should “allow some level of stability first and life-saving and immediate needs [to be responded to]” before involving children in data collection. Other respondents often agreed with the general sentiment that the early phases of emergencies could present high risks to children’s safe participation, especially in contexts of ongoing conflict, but they did not feel that these conditions should categorically preclude participation. As one respondent wrote, “[i]t’s not appropriate to interview children in any context if the researchers don’t have the protective/ethical procedures in place and necessary skills and contingency follow-up plans. However, if they do, then it should be appropriate to collect data from children in the early phases of an emergency.”

### How should child participants be selected?

#### Individual characteristics and experiences

Almost all respondents agreed that “clear and transparent” inclusion and exclusion criteria should be developed for selection of child participants (98%, S86) and that the principle of justice should underpin all assessment activities, which includes efforts to make participant selection equitable and fair (95%, S14). Respondents described a common practice of interviewing boys more than girls, and affirmed that girls and boys should participate in data collection activities equally (95%, S89). The majority of respondents also noted that clan and tribal associations were important factors in selecting participants in emergency settings (90%, S88).

There was much less agreement about how other individual attributes should factor into participant selection. For example, none of the eight statements related to the role of age in determining a child’s eligibility achieved clear consensus. Several respondents provided minimum ages at which they thought children should be eligible to participate, though the proposed minimum age varied widely, from 5 years (80%, S66) to 16 years (10%, S72). Others felt that age should not determine a child’s eligibility in itself. As one respondent wrote, “I find it difficult to respond to these age-related questions, since age is something relative and is strongly contextually and culturally dependent.” Another respondent noted that eligibility should be determined by the “evolving capacities of the child,” emphasizing that the importance of a child’s competencies and life experiences should be taken into account in addition to the child’s age.

Respondent opinions were decidedly mixed about how particular experiences should factor into participant eligibility. For example, a little over half of respondents felt that a child should not participate in data collection if the child had suffered from a traumatic incident in the recent past (55%, S19). Others, though, thought child involvement depended on the context, the competency of the team, the methods, the purpose of the data collection activity, and the availability of services. Less than two-thirds of respondents agreed that special efforts should be made to involve the most vulnerable child populations in data collection activities, such as children with disabilities, outside of family care, or in conflict with the law, but with the caveat that these populations should be excluded if specialized services were not available to treat them (63%, S87). As one respondent elaborated, “There are many settings where services will not be put in place until there is evidence of the need for those services. Systematically excluding sensitive topics or particular groups of children needs further examination.”

#### Presence, consent, and involvement of caregiver

A sizeable minority of respondents felt that data should only be collected from children with parental consent and involvement (29%, S61), though a few of these respondents also noted that exceptions could be made for older children or in cases where children were outside of family care. According to one respondent, involvement of a caregiver “depends on the age and maturity of the child. There may be instances where children are no longer with their parents.” A number of others wrote similar remarks, while also adding that parental involvement can influence child testimony in some cases or increase the risk of harm. As one respondent noted, the involvement of parents “needs further consideration, especially when the subject matter is related to how [children] are being treated by their parents and/or could put children in a harmful situation.”

One in ten respondents felt that adult caregivers should be present during data collection with children, though the majority of respondents strongly believed a caregiver need not always be present (DI = 0.81, S78). At the same time, most respondents agreed that the presence of trusted adults might help make children feel secure, so long as the child had the choice about whether or not he or she wanted to participate (93%, S80). A small fraction (7%) of respondents also felt that children separated from caregivers should not be included in data collection, though the majority of respondents disagreed with this statement, and often strongly (DI = 0.84, S63).

### How should children participate in emergency assessments and related data collection activities?

#### Child-friendly methods

Respondents came to clear consensus on 10 of 14 statements related to child-friendly methods, with an additional two statements approaching consensus (see Tables 2 and 3). All respondents agreed that data collectors should adapt their methods to children’s age, capacities, cultures, and languages (S93, S94, S99). Most respondents agreed that a critical component of a child-friendly approach was to try to minimize power differentials between investigators and child participants (93%, S106), while also managing power dynamics among child participants (100%, S85). Respondents also agreed that all tools should undergo cognitive and field testing ahead of data collection (98%, S95), that investigators should identify private and secure locations for children to participate (100%, S102), and that data collection activities should allow sufficient time for children to participate meaningfully (98%, S98) while still accommodating children’s other time obligations (100%, S96). Respondents were also unified in emphasizing that informed consent was an ongoing process that should be re-evaluated continuously throughout the data collection process (95%, S58).

#### Sensitive topics

There were important points of contention about whether certain topical issues should be excluded from data collection exercises with children. Almost half (48%) of respondents thought that all topics could be explored with child participants, while about a quarter (26%) disagreed, and another quarter (26%) was undecided (DI = 0.5, S17). Several of those who agreed that all topics could be explored qualified their statements by saying that sensitive topics should be explored only as long as investigators could safeguard participants, secure their confidentiality, and adapt the data collection methods to their context and capacities. As one respondent explained, “[i]t isn’t the subject that determines whether it is appropriate to involve children, but the processes used and the context in which the information-gathering takes place.”

Other respondents felt that the risks associated with discussing certain topics with children were simply too high in some contexts, regardless of the safeguards in place. As one respondent related, “sex is still a taboo in many settings for children to freely talk about; young children seen to be more knowledgeable on sex matters are considered immoral and unfit in some communities.” Another respondent believed that asking children about possible exposure to sexual violence was especially inappropriate. The respondent reasoned that, in these cases, “[i]nterviewing children on what happened to them would potentially cause a lot of harm.” In a related vein, over half of respondents agreed that, “questions that dig into personal experiences, particularly negative emergency-related experiences, should be avoided” (59%, S18). Those who disagreed with this latter statement sometimes specified that highly trained investigators, such as psychologists and social workers, could engage children on these topics in safe, meaningful, and beneficial ways.

Several respondents noted the need for additional guidance and positive examples of data collection on sensitive topics. As one respondent remarked, it “[w]ould be especially helpful to ensure these examples are documented and widely shared so that others are able to replicate work with such sensitive issues in as responsible and ethical a manner as possible. Otherwise this can be VERY dangerous ground to tread. Unfortunately even in recent times I’ve seen numerous harmful examples in the field.”

Two points of clear consensus for reducing the risk of harm included the need to secure a protective environment and support network (95%, S21) and the need to consult with local leaders and specialists (90%, S22) before broaching sensitive topics with children.

#### Communicating expectations and results

Respondents unanimously agreed that investigators had a responsibility to communicate and manage expectations with participants before, during, and after assessments and other evidence generation activities (100%, S59; 98%, S60), but they did not always agree about how the findings should be relayed to child participants. Although 93% of respondents felt that children should receive feedback on the findings of assessments, and should continue to be involved after data collection has stopped (S118), only 80% agreed that staff should return after data collection to review the findings with participating children, and make adjustments according to feedback (S117). Dissenters explained that returning to the same individuals would be challenging for logistics and security reasons, while also noting that involving the same group of participants more than once could “over-burden” those children or create a perception of favoritism. There was even less agreement as to whether children and their parents should be able to see the results of data collection immediately and be invited to modify study results (55%, S116). A common reason for disagreeing was that sharing findings with parents is often inappropriate, especially if they are also given a chance to modify the results. There was clear consensus that findings that “call into question the actions of adults” should be handled sensitively so as not to provoke retaliation against children (90%, S119).

## Discussion

The findings from this Delphi review have several important implications for standard setting and coordination within the CPiE sector. Respondents clearly valued the principle of children’s participation in data collection activities during emergencies, as well as the basic principles of research ethics, including respect for persons, beneficence, and justice. They also largely agreed on a number of ‘good practices’ for involving children. These included, for example, assessing competencies of data collection staff, conducting risk analyses, requesting informed consent continuously, creating clear protocols for data collection—with explicit selection criteria and referral plans—and adapting and field-testing instruments to ensure they are suitable for the selected participants. While these sentiments shared broad support, none of these activities are insignificant when undertaken in emergency contexts, and findings may indicate the need for shifts in current practice. The findings also reveal points of ongoing debate, uncertainty, and ambivalence within the CPiE community that merit attention as they may contribute to inconsistent data collection practice [10, 25].

### Staff competencies

The clear consensus around the need for investigators that are qualified to work with children raises questions around how to define minimum competencies, and how to build these capacities ahead of emergency events. The perceived unavailability of qualified data collection staff is a principal reason for discouraging child involvement in the Child Protection Rapid Assessment, a premise that has been supported by a subsequent review of the CPRA’s use in 15 countries, and yet the sector has no shared standard for assessing staff capacities to work with children [24, 25]. In recent years, UN agencies and NGOs have led numerous efforts to improve CPiE evidence generation capacities through training activities and academic-practitioner collaborations. The Alliance for Child Protection in Humanitarian Action and the Child Protection Area of Responsibility, for example, have conducted eight ‘training of trainers’ sessions around the globe to increase the pool of practitioners capable of leading CPRAs, though these trainings do not include techniques for engaging children directly. Meanwhile, the University of Kwazulu-Natal hosts a distance-learning CPiE Postgraduate Diploma program through a partnership between the Alliance for Child Protection in Humanitarian Action, UNICEF, and Save the Children UK [38]. Additionally, the Center on Child Protection and Wellbeing (PUSKAPA) at the University of Indonesia offers a child protection specialization for master’s students while also training civil society partners on best practices for engaging children in research [39].

These initiatives and models for capacity building are all relatively new, and their ability to ensure that highly qualified staff are available to collect data with children in emergencies has yet to be established. The findings of this study reflect a critical need to continue investing in capacity-building strategies, and to ensure that these efforts include dedicated components for addressing children’s participation in data collection activities.

### Using risk analyses to determine whether children should participate

While almost all respondents agreed that decisions about whether children should participate in a given data collection activity should be determined by a contextual risk analysis rather than by predetermined ‘rules of thumb,’ respondents often disagreed about how political, cultural, environmental, social, economic, and security variables should factor into these decisions. It is evident from these findings that, given the same scenarios and information, CPiE specialists in this study would sometimes arrive at different decisions about whether to involve children. For instance, 70% of respondents felt that interviewing children was still important when information from other sources was available, and 43% said that they would not interview children in the early phases of emergencies if basic services were unavailable or if there was still a significant incidence of violence targeting children. These unexpected disagreements are indicative less of contextual decision-making than of discordance in the application of the participation principle. They also signal a divide between practitioner opinion and some of the existing guidelines on child participation, which list the availability of basic services and the unavailability of alternative data sources as minimum criteria for children’s participation [10, 13, 20].

Inconsistency in decision-making about whether children participate in emergency-related data collection activities can have serious consequences for children and their communities, as well as for the validity of the data collected. One study, for example, found that CPRAs in several countries involved children as participants despite the CPRA guideline’s recommendation not to [25]. While child participants expressed gratitude for being included in some countries, involvement with inadequate safeguards in place reportedly caused harm to the participants in at least one context. Given this potential for harm, the findings reflect an urgent need for more deliberation and consensus building around how to identify and weigh risks into child participation decisions, especially around divisive issues, such as the relevance of the type and phase of emergency.

### Determining eligibility based on the evolving capacities of the child

The discrepancies in respondent opinions about how to determine participant eligibility also prompt reflection. It is especially important to note that the range of minimum age requirements that respondents proposed, and often agreed to, conflicts with existing standards, which recommend that age be just one factor in determining a child’s capacity to participate, together with their life experiences and competencies [7, 21]. It was also surprising that almost half of respondents felt that recent exposure to a trauma should categorically preclude a child from participating, as screening for exposure to traumatic experiences requires a specialized skill set that is usually unavailable in humanitarian settings. The study group’s ambivalence about how to determine eligibility for child participants with special needs in the absence of specialized services warrants further attention as well. These questions have critical implications for the principles of justice and non-maleficence, and for securing useful data to inform programs targeting vulnerable child populations in emergencies [25].

### Studying sensitive topics with children

Although respondents reached consensus about a range of appropriate methods and techniques for engaging children in safe and meaningful data collection, how to investigate sensitive topics was a notable exception. Given the fact that interviewing children directly about sensitive topics, such as exposure to sexual violence and involvement with armed groups, may pose unique risks to children, it is highly significant that about half of the respondents thought certain topics should not be broached with children while the other half disagreed [13, 20, 21]. A previous survey, which was not limited to emergency contexts, found that researchers’ concern about whether certain topics are too sensitive to include in child interviews varied widely, with researchers from lower- and middle-income countries often being more concerned about the sensitivity of the topic than those from higher income countries [40]. This disparity likely affects the ability for organizations to collect certain types of data in many humanitarian settings. Yet, as several respondents remarked, there is little guidance on identifying topics that could be sensitive and on deciding whether to include these topics in data collection involving children [16, 41]. Respondents did not delineate how investigators should weigh children’s confidentiality against the imperative to report abuses exposed through data collection activities, but this is certainly another important ethical consideration that merits further investigation [10, 12].

### Limitations

This study is not without limitations. We purposively sampled specialists via multiple channels in order to capture a diverse array of perspectives on the research questions. Nevertheless, certain groups may have been overrepresented, while others, especially donor representatives and specialists from South and Central America, Australasia, and the Middle East, were underrepresented. Another limitation of the study was the length and complexity of statements generated for Rounds II and III. While analyzing the Round I questionnaires, we made every effort to preserve the nuances of meaning and original phrasing of the responses while also providing clarity. This had the effect of generating statements with numerous clarifying clauses, making them highly specific. Respondents frequently reported agreeing with only fractions of statements, or feeling uncomfortable agreeing with a statement without adding further caveats, also remarking that their opinions were context-dependent. For the 17 statements that approached consensus without achieving clear consensus, participants often either considered them to be worded too vaguely or too restrictively (Table 3). Respondents also commented that the Round II and Round III survey were long, which in one case led to an incomplete survey and in a few others likely contributed to attrition.

## Conclusion

Despite its limitations, this study provides valuable insights for child protection practitioners, researchers, donors, and policymakers who work in humanitarian contexts. The study reflects notable coherence among specialists not only in their appreciation for general research and child rights principles, but also in their identification of the risks that data collection activities present to children, and approaches to mitigating those risks. Points of ongoing debate around how to factor different risks—including the phase of emergency, the existence of basic services, and children’s recent exposure to trauma—into child participation decisions may be more complicated to resolve, but warrant attention. Further engagement with these unresolved questions is needed within the CPiE sector to uphold the participation principle and ensure children’s safe and meaningful engagement in emergency-related data collection activities.

## Additional file

Additional file 1 All Delphi Statements with Final Consensus Ratings, by Theme. (XLSX 57 kb)

## Abbreviations

AI: Agreement intensity
CPiE: Child protection in emergencies
CPRA: Child protection rapid assessment
CPWG: Child protection working group
CRC: Convention on the rights of the child
DI: Disagreement intensity
IRB: Institutional review board
MIRA: Multi cluster/sector initial rapid assessment
PUSKAPA: Pusat Kajian & Advokasi Perlindungan & Kualitas Hidup Anak (Center on Child Protection and Wellbeing)
UN: United Nations

## References

[1] Committee on the Rights of the Child (CRC). Convention on the rights of the child. General comment no. 12: The right of the child to be heard; 2009.

[2] Beazley H, Bessell S, Ennew J, Waterson R (2009). The right to be properly researched: Research with children in a messy, real world. Child Geographies.

[3] Boyden, J, Ennew, J. Children in focus: a manual for participatory research with children. Stockholm: Save the Children Sweden; 1997.

[4] Cahill, H, Beadle, S, Mitch, J, Coffey, J, Crofts, J. Adolescents in emergencies. Parkville: University of Melbourne; 2010.

[5] Dyregrov K, Dyregrov A, Raundalen M (2010). Refugee families’ experience of research participation. J Trauma Stress.

[6] Gibbs L, Mutch C, O’Connor P, MacDougall C (2013). Research with, by, for and about children: Lessons from disaster contexts. Global Studies Child.

[7] Graham A, Powell M, Taylor N, Anderson D, Fitzgerald R (2013). Ethical research involving children.

[8] Thomas S, Byford S (2003). Research with unaccompanied children seeking asylum. Br Med J.

[9] Vervliet, M, Rousseau, C, Broekaert, E, Derluyn, I. Multilayered Ethics in Research Involving Unaccompanied Refugee Minors. J Refugee Studies. 2015; doi: 10.1093/jrs/feu039.

[10] Berman G, Hart J, O’Mathúna D, Mattellone E, Potts A, O’Kane C, et al. What we know about ethical research involving children in humanitarian settings: an overview of principles, the literature and case studies (Innocenti Working Paper, No. 2016-18). Florence: UNICEF Office of Research; 2016.

[11] Feinstein C, O’Kane C (2008). A kit of tools for participatory research and evaluation with children, young people and adults: A compilation of tools used during a thematic evaluation and documentation on children’s participation in armed conflict, post conflict and peace building.

[12] Feinstein C, O’Kane C (2008). Ethical guidelines for ethical, meaningful and inclusive children’s participation practice.

[13] International Rescue Committee (IRC) (2012). Caring for child survivors of sexual abuse: Guidelines for health and psychosocial service providers in humanitarian settings.

[14] Lansdown G, O’Kane C (2014). A toolkit for monitoring and evaluating children’s participation.

[15] Mann, G, Tolfree, D. Children’s participation in research: Reflections from the care and protection of separated children in emergencies project. Stockholm: Save the Children Sweden; 2003.

[16] O’Kane, C. Guidelines for children’s participation in humanitarian programming. London: Save the Children; 2013.

[17] Sumathipala, A, Jafarey, A, de Castro, L, Ahmad, A, Marcer, D, Srinivasan, S, et al. The draft statement/guidelines for disaster research, working group on disaster research and ethics. Global Health Trials. 2011, Available: https://globalhealthtrials.tghn.org/articles/draft-statementguidelines-disaster-research/. Accessed 25 October 2016.

[18] Toms, C, MacLeod, H. Children in emergencies manual. Monrovia, CA: World Vision International; 2006.

[19] UNICEF (2007). The participation of children and young people in emergencies: A guide for relief agencies, based largely on experiences in the Asian tsunami response.

[20] World Health Organization (WHO) (2007). WHO ethical and safety recommendations for researching, documenting and monitoring sexual violence in emergencies.

[21] Hart J, Tyrer B (2006). Research with children living in situations of armed conflict: Concepts, ethics and methods.

[22] Jacobsen K, Landau L (2003). B. The dual imperative in refugee research: some methodological and ethical considerations in social science research on forced migration. Disasters.

[23] Inter-Agency Standing Committee. (IASC) (2012). Multi-cluster/sector initial rapid assessment (MIRA): Provisional version March 2012.

[24] Child Protection Working Group (CPWG). Child protection rapid assessment toolkit. Global Protection Cluster; 2012.

[25] Landis D, Stark L, Mansourian H, Ager A (2013). Examining Child Protection Rapid Assessment: A structured review of field learning from the Child Protection Rapid Assessment (CPRA) toolkit.

[26] Alliance for Child Protection in Humanitarian Action. Assessment, measurement and evidence working group. 2017; Available: https://alliancecpha.org/assessment-and-measurement-working-group/. Accessed 3 January, 2017.

[27] Dalkey NC, Brown BB, Cochran S (1969). The Delphi method: An experimental study of group opinion (Vol. 3).

[28] Ager A, Stark L, Akesson B, Boothby N (2010). Defining best practice in care and protection of children in crisis affected settings: A delphi study. Child Dev.

[29] Powell C (2003). The Delphi technique: myths and realities. J Adv Nurs.

[30] Beattie E, Mackway-Jones K (2004). A Delphi study to identify performance indicators for emergency medicine. Emerg Med J.

[31] Brown N, Crawford I, Carley S, Mackway-Jones K (2006). A Delphi-based consensus study into planning for biological incidents. J Public Health.

[32] Crawford IWF, Mackway-Jones K, Russell DR, Carley SD (2004). Planning for chemical incidents by implementing a Delphi based consensus study. Em Med J.

[33] Walker AM, Selfe J (1996). The Delphi method: a useful tool for the allied health researcher. Int J Ther Rehabil.

[34] Hasson F, Keeney S, McKenna H (2000). Research guidelines for the Delphi survey technique. J Adv Nurs.

[35] Hasson F, Keeney S (2011). Enhancing rigour in the Delphi technique research. Technol Forecast Soc Chang.

[36] Hsu CC, Sandford BA (2007). The Delphi technique: making sense of consensus. Pract Assessment Res Eval.

[37] Braun V, Clarke V, Terry G (2014). Thematic analysis. Qual Res Clin Health Psychol.

[38] Child Protection Working Group (CPWG). CPiE diploma. 2016; Available: http://cpwg.net/what-we-do/capacity-building/cpie-diploma/. Accessed 25 Oct 2016.

[39] Pusat Kajian & Advokasi Perlindungan & Kualitas Hidup Anak. Child protection graduate program. 2016; Available: http://puskapa.org/. Accessed 25 Oct 2016.

[40] Powell, M A, Graham, A, Taylor, N J, Newell, S, Fitzgerald, R. Building capacity for ethical research with children and young people: An international research project to examine the ethical issues and challenges in undertaking research with and for children in different majority and minority world contexts (Research Report for the Childwatch International Research Network). Dunedin: University of Otago Centre for Research on Children and Families/Lismore: Centre for Children and Young People; 2011.

[41] Schenk K, Williamson J (2005). Ethical approaches to gathering information from children and adolescents in international settings: Guidelines and resources.

